# Racial/ethnic and geographic differences in second primary cancers in stomach cancer survivors: a comparative study of U.S. and South Korea

**DOI:** 10.1007/s10120-026-01728-9

**Published:** 2026-03-09

**Authors:** Yuntong Wang, Dong-Woo Choi, Sangwon Lee, Jialin Mao, Art Sedrakyan, Xiang Shu, Kui Son Choi, Heejung Chae, Eunji Choi

**Affiliations:** 1 Department of Population Health Sciences, Weill Cornell Medicine, 425 E 61st Street, New York, NY 10065 USA; 2 National Cancer Control Institute, National Cancer Center, Goyang-si, Gyeonggi-do South Korea; 3 Department of Epidemiology and Biostatistics, Memorial Sloan Kettering Cancer Center, New York, NY USA; 4 National Cancer Center Graduate School of Cancer Science and Policy, National Cancer Center, Goyang, South Korea; 5 Seoul National University Bundang Hospital, Seongnam, Republic of Korea

**Keywords:** Second primary cancer, Multiple primary cancer, Racial/ethnic disaprity, Epidemiology, Incidence, Mortality

## Abstract

**Background:**

Stomach cancer remains the fifth leading cause of cancer death, with racial/ethnic and geographic differences. As survival improves, these survivors face elevated risks of second primary cancers (SPCs). We evaluated SPC risk and post-SPC survival by race/ethnicity and between the U.S. and South Korea.

**Methods:**

We analyzed patients with stage I–III stomach cancer (2012–2020) from the U.S. SEER-17 and Korea Cancer Public-Library Database. SPCs were defined as non-gastric cancers diagnosed > 1 year post initial diagnosis. Cumulative SPC incidence was estimated using Aalen-Johansen method to account for competing death. Cox regression assessed survival impact of SPC development.

**Results:**

Among 19,595 U.S. and 204,240 Korean patients, 1,050 and 6,908 developed SPCs, respectively. In the U.S., 5-year SPC incidence was highest in Black (7.4% [6.1–8.6]) and lowest in Pacific Islanders (4.5% [2.3–6.7]). Notable heterogeneity in SPC incidence was observed across Asian subgroups, with Korean Americans showing the highest (6.9% [4.7–9.1]) and Filipinos the lowest (4.4% [1.9–7.2]). In contrast, Korean survivors in South Korea had the lowest SPC incidence (3.8% [3.7–3.9]). SPC development was associated with higher mortality (HR = 2.22 [1.96–2.52]), with a stronger effect in Asians (HR = 3.12) than Whites (HR = 1.96; interaction *P* < 0.1). Among Asian subgroups, SPC development was associated with the highest mortality in Vietnamese (HR = 13.6) and the lowest in Filipino survivors (HR = 1.54; interaction *P* < 0.01). Among Korean survivors in South Korea, SPC development conferred a modest increase in mortality risk (HR = 1.68 [1.61–1.75]).

**Conclusions:**

SPC risk and outcomes in stomach cancer survivors vary by race/ethnicity and country, particularly within Asian subgroups.

**Supplementary Information:**

The online version contains supplementary material available at 10.1007/s10120-026-01728-9.

## Introduction

Stomach cancer remains a global health burden, ranking as the fifth most common malignancy worldwide, with an estimated 968,784 new cases and 660,175 deaths annually. [1, 2] Early endoscopic detection and treatment advances have improved stomach cancer survival from 15% to 35.8% (1975–2024) in the U.S. [3] and from 55.7% to 77.0% (1999–2019) in South Korea. [4] With improved long-term survival, these stomach cancer survivors face increasing risks of second primary cancers (SPCs). [5, 6] Given the increasing burden of SPCs among stomach cancer survivors, a few country-specific studies have assessed SPC burden relative to the cancer-free general population using standardized incidence ratios. [7–10] However, there remains a critical gap in SPC epidemiology by race/ethnicity using cross-national comparisons, as well as survival outcomes specifically attributable to SPC development.

Racial/ethnic and geographic differences in initial primary stomach cancer incidence and mortality are well recognized. Asian Americans consistently experience elevated stomach cancer rates in the U.S. [11–16] However, data on racial/ethnic and geographic differences in SPC risk and post-SPC survival outcomes have been lacking in stomach cancer survivors. Most epidemiologic studies continue to group Asians as a single population, obscuring important differences in tumor characteristics, risk profiles, and clinical outcomes across subgroups. [17–19] Cross-national comparisons incorporating detailed racial and geographic stratification are also limited, yet they are essential for identifying individual- and system-level factors underlying these differences.

We used population-based data from the U.S. Surveillance, Epidemiology, and End Results (SEER) Program and the South Korea Cancer Public Library Database (CPLD) to estimate SPC risk and evaluate post-SPC survival among stomach cancer survivors. Our analyses focused on racial/ethnic and geographic differences, with particular attention to disaggregated Asian subgroups, to advance understanding of long-term cancer outcomes in diverse populations.

## Methods

### Study population and data source

This study used two national cancer registries: the U.S. SEER-17 registries and the Cancer Public Library Database (CPLD) in South Korea. [20] Both databases provide patient demographics, tumor characteristics, first-course treatment, and survival outcomes. Detailed descriptions are in the Supplementary Methods.

Our study cohort included individuals diagnosed with primary invasive stomach cancer between 1/1/2012, and 12/31/2020. Given the limited relevance of SPCs among short-term survivors, we restricted the analysis to patients who survived at least 12 months after diagnosis. Patients with distant-stage disease were also excluded due to high competing mortality and low expected SPC risk. We also excluded cases with unknown race/ethnicity or missing key variables such as survival time. To ensure consistency, identical inclusion and exclusion criteria were applied to patients in the Korea CPLD (Supplementary Fig. 1).

### Definition of second primary cancer (SPC)

The U.S. SEER and Korea CPLD capture only new primary cancers. [21] Yet, to minimize the misclassification bias between new SPCs vs. recurrences, we defined SPCs as non-stomach malignancies diagnosed at least 12 months after the initial stomach cancer diagnosis. We also did not consider non-melanoma skin cancer and basal cell skin cancer as SPCs, which are almost not lethal.

### Primary outcomes

We evaluated two primary outcomes. The first was the 5-year cumulative incidence of SPCs. The second primary outcome was overall mortality. Survival time was defined as the interval from the date of initial stomach cancer diagnosis to the date of all-cause death, last documented follow-up, or the administrative censoring date (12/31/2021). Outcomes were analyzed by race/ethnicity—including disaggregated Asian subgroups in the U.S.—and by country (U.S. vs. South Korea).

### Statistical analysis

*Cumulative Incidence of SPCs.* The Aalen-Johansen estimator was employed to estimate the cumulative risk of SPC over time while accounting for high competing risk of death in stomach cancer survivors. [22, 23] Gray’s test was used to compare cumulative incidence curves across racial/ethnic subgroups to evaluate statistically significant differences. [24, 25] .

*Survival Impact of SPC Development.* A multivariable Cox proportional hazards model was used to estimate adjusted hazard ratios (HRs) for overall mortality associated with SPC development. SPC status was modeled as a time-varying covariate to account for its occurrence during follow-up and to prevent immortal time bias. [26] The model was adjusted for key prognostic factors and confounders, including patient demographics, tumor characteristics, and treatment. We conducted subgroup analyses by race/ethnicity and disaggregated Asian subgroup (Chinese, Korean, Japanese, Vietnamese, Filipino) in the U.S. SEER data. A likelihood ratio test was conducted to assess the statistical difference in SPC’s survival impact on mortality by race/ethnicity and by Asian subgroups.

*Exploratory Analysis of Potential Determinants.* To explore factors underlying differences in SPC risk and mortality by race/ethnicity and country, we examined common SPC types, age, stage, tumor size, and time to treatment. Furthermore, since radiotherapy for the initial cancer has long been considered a major factor contributing SPC risk for various cancer survivors, [27–32] we further estimated cumulative SPC incidence by initial radiotherapy status.

## Results

A total of 19,595 stomach cancer survivors in the U.S. cohort, 1,050 (5.4%) developed SPCs during follow-up. The highest proportion of SPC was observed in White patients (6.2%), followed by Black (5.8%), Asian (4.7%), Latino (3.8%), and Pacific Islander (3.3%) patients (Table 1). Within Asian subgroups, SPC proportions were highest in Korean American patients (5.3%), followed by Japanese (5.1%), Chinese (4.8%), Filipino (4.4%), and Vietnamese (3.9%) American patients (Table 1). In the South Korean cohort of 204,240 stomach cancer patients, 6,908 (3.4%) developed SPCs (Table 2).

**Table 1:** Patient and tumor characteristics of stomach cancer survivors by race and Asian subgroup in the U.S

	By race/ethnicity					By asian subgroup				
	White	Latino	Black	Asian	Pacific Islander	Korean	Chinese	Japanese	Filipino	Vietnamese
	*N* = 9,681	*N* = 3,949	*N* = 2,590	*N* = 2,743	*N* = 632	*N* = 756	*N* = 728	*N* = 350	*N* = 344	*N* = 305
*Development of second primary cancer during follow-up*										
Yes	601 (6.2%)	150 (3.8%)	150 (5.8%)	128 (4.7%)	21 (3.3%)	40 (5.3%)	35 (4.8%)	18 (5.1%)	15 (4.4%)	12 (3.9%)
*Vital status*										
No	6,010 (62.1%)	2,499 (63.3%)	1,692 (65.3%)	1,798 (65.5%)	462 (73.1%)	524 (69.3%)	465 (63.9%)	187 (53.4%)	227 (66.0%)	200 (65.6%)
Yes	3,671 (37.9%)	1,450 (36.7%)	898 (34.7%)	945 (34.5%)	170 (26.9%)	232 (30.7%)	263 (36.1%)	163 (46.6%)	117 (34.0%)	105 (34.4%)
*Follow-up time*										
Mean (SD)	3.1 (± 2.5)	2.9 (± 2.5)	3.1 (± 2.5)	3.3 (± 2.5)	3.2 (± 2.5)	3.4 (± 2.6)	3.1 (± 2.5)	3.3 (± 2.6)	3.2 (± 2.5)	3.2 (± 2.6)
*Sex*										
Female	3,675 (38.0%)	1,906 (48.3%)	1,292 (49.9%)	1,157 (42.2%)	259 (41.0%)	285 (37.7%)	318 (43.7%)	168 (48.0%)	178 (51.7%)	117 (38.4%)
Male	6,006 (62.0%)	2,043 (51.7%)	1,298 (50.1%)	1,586 (57.8%)	373 (59.0%)	471 (62.3%)	410 (56.3%)	182 (52.0%)	166 (48.3%)	188 (61.6%)
*Age at stomach cancer diagnosis*										
<40	232 (2.4%)	295 (7.5%)	109 (4.2%)	69 (2.5%)	36 (5.7%)	9 (1.2%)	19 (2.6%)	3 (0.9%)	14 (4.1%)	7 (2.3%)
40–49	616 (6.4%)	515 (13.0%)	264 (10.2%)	204 (7.4%)	61 (9.7%)	42 (5.6%)	60 (8.2%)	11 (3.1%)	25 (7.3%)	26 (8.5%)
50–59	1,768 (18.3%)	900 (22.8%)	596 (23.0%)	394 (14.4%)	141 (22.3%)	94 (12.4%)	99 (13.6%)	27 (7.7%)	53 (15.4%)	66 (21.6%)
60–69	3,009 (31.1%)	1,037 (26.3%)	812 (31.4%)	800 (29.2%)	173 (27.4%)	240 (31.7%)	211 (29.0%)	73 (20.9%)	109 (31.7%)	86 (28.2%)
70–79	2,648 (27.4%)	800 (20.3%)	553 (21.4%)	804 (29.3%)	156 (24.7%)	264 (34.9%)	200 (27.5%)	110 (31.4%)	98 (28.5%)	85 (27.9%)
80+	1,408 (14.5%)	402 (10.2%)	256 (9.9%)	472 (17.2%)	65 (10.3%)	107 (14.2%)	139 (19.1%)	126 (36.0%)	45 (13.1%)	35 (11.5%)
*SEER summary stage of initial stomach cancer*										
Localized	5,774 (59.6%)	2,154 (54.5%)	1,582 (61.1%)	1,469 (53.6%)	373 (59.0%)	449 (59.4%)	352 (48.4%)	186 (53.1%)	192 (55.8%)	136 (44.6%)
Regional	3,907 (40.4%)	1,795 (45.5%)	1,008 (38.9%)	1,274 (46.4%)	259 (41.0%)	307 (40.6%)	376 (51.6%)	164 (46.9%)	152 (44.2%)	169 (55.4%)
*Histological type of initial stomach cancer*										
Diffuse type	1,252 (12.9%)	900 (22.8%)	304 (11.7%)	634 (23.1%)	123 (19.5%)	198 (26.2%)	162 (22.3%)	69 (19.7%)	90 (26.2%)	69 (22.6%)
Intestinal type	5,152 (53.2%)	1,787 (45.3%)	1,156 (44.6%)	1,517 (55.3%)	318 (50.3%)	463 (61.2%)	427 (58.7%)	223 (63.7%)	121 (35.2%)	173 (56.7%)
NOS/Other	3,277 (33.8%)	1,262 (32.0%)	1,130 (43.6%)	592 (21.6%)	191 (30.2%)	95 (12.6%)	139 (19.1%)	58 (16.6%)	133 (38.7%)	63 (20.7%)
*Anatomic site of initial stomach cancer*										
Cardia	4,027 (41.6%)	586 (14.8%)	286 (11.0%)	323 (11.8%)	94 (14.9%)	43 (5.7%)	77 (10.6%)	56 (16.0%)	74 (21.5%)	24 (7.9%)
Distal	1,256 (13.0%)	915 (23.2%)	669 (25.8%)	877 (32.0%)	170 (26.9%)	283 (37.4%)	246 (33.8%)	101 (28.9%)	66 (19.2%)	126 (41.3%)
Middle	2,764 (28.6%)	1,568 (39.7%)	1,018 (39.3%)	1,095 (39.9%)	235 (37.2%)	317 (41.9%)	291 (40.0%)	140 (40.0%)	143 (41.6%)	102 (33.4%)
Overlapping/Other	1,634 (16.9%)	880 (22.3%)	617 (23.8%)	448 (16.3%)	133 (21.0%)	113 (14.9%)	114 (15.7%)	53 (15.1%)	61 (17.7%)	53 (17.4%)
*Radiotherapy*										
No	6,744 (69.7%)	3,144 (79.6%)	2,116 (81.7%)	2,213 (80.7%)	524 (82.9%)	652 (86.2%)	575 (79.0%)	266 (76.0%)	279 (81.1%)	234 (76.7%)
Yes	2,937 (30.3%)	805 (20.4%)	474 (18.3%)	530 (19.3%)	108 (17.1%)	104 (13.8%)	153 (21.0%)	84 (24.0%)	65 (18.9%)	71 (23.3%)
*Chemotherapy*										
No	4,833 (49.9%)	1,899 (48.1%)	1,318 (50.9%)	1,319 (48.1%)	319 (50.5%)	418 (55.3%)	341 (46.8%)	168 (48.0%)	136 (39.5%)	133 (43.6%)
Yes	4,848 (50.1%)	2,050 (51.9%)	1,272 (49.1%)	1,424 (51.9%)	313 (49.5%)	338 (44.7%)	387 (53.2%)	182 (52.0%)	208 (60.5%)	172 (56.4%)
*Surgery*										
No	2,016 (20.8%)	763 (19.3%)	475 (18.3%)	389 (14.2%)	115 (18.2%)	97 (12.8%)	90 (12.4%)	57 (16.3%)	52 (15.1%)	51 (16.7%)
Yes	7,665 (79.2%)	3,186 (80.7%)	2,115 (81.7%)	2,354 (85.8%)	517 (81.8%)	659 (87.2%)	638 (87.6%)	293 (83.7%)	292 (84.9%)	254 (83.3%)
*Tumor size of initial stomach cancer at detection*										
<2 cm	2,299 (23.7%)	880 (22.3%)	515 (19.9%)	565 (20.6%)	138 (21.8%)	210 (27.8%)	129 (17.7%)	72 (20.6%)	53 (15.4%)	47 (15.4%)
2-<4 cm	2,168 (22.4%)	815 (20.6%)	532 (20.5%)	766 (27.9%)	155 (24.5%)	216 (28.6%)	202 (27.7%)	108 (30.9%)	94 (27.3%)	84 (27.5%)
4-<6 cm	1,511 (15.6%)	660 (16.7%)	510 (19.7%)	475 (17.3%)	99 (15.7%)	102 (13.5%)	145 (19.9%)	53 (15.1%)	65 (18.9%)	74 (24.3%)
6-<10 cm	1,044 (10.8%)	507 (12.8%)	359 (13.9%)	375 (13.7%)	83 (13.1%)	79 (10.4%)	101 (13.9%)	51 (14.6%)	59 (17.2%)	49 (16.1%)
>=10 cm	436 (4.5%)	227 (5.7%)	211 (8.1%)	145 (5.3%)	46 (7.3%)	25 (3.3%)	46 (6.3%)	14 (4.0%)	35 (10.2%)	16 (5.2%)
Missing	2,223 (23.0%)	860 (21.8%)	463 (17.9%)	417 (15.2%)	111 (17.6%)	124 (16.4%)	105 (14.4%)	52 (14.9%)	38 (11.0%)	35 (11.5%)
*Time to treatment (months)*										
Mean (SD)	1.3 (± 1.5)	1.5 (± 1.9)	1.3 (± 1.7)	1.4 (± 1.5)	1.4 (± 1.7)	1.5 (± 1.8)	1.3 (± 1.3)	1.4 (± 1.3)	1.5 (± 1.9)	1.3 (± 1.2)
Missing	941 (9.7%)	513 (13.0%)	266 (10.3%)	359 (13.1%)	100 (15.8%)	136 (18.0%)	75 (10.3%)	41 (11.7%)	31 (9.0%)	41 (13.4%)
H*ousehold income*										
<60k	1,704 (17.6%)	365 (9.2%)	665 (25.7%)	44 (1.6%)	19 (3.0%)	5 (0.7%)	7 (1.0%)	5 (1.4%)	4 (1.2%)	11 (3.6%)
60k–75k	2,578 (26.6%)	1,388 (35.1%)	844 (32.6%)	603 (22.0%)	159 (25.2%)	226 (29.9%)	140 (19.2%)	62 (17.7%)	65 (18.9%)	50 (16.4%)
75k–90k	2,427 (25.1%)	1,172 (29.7%)	609 (23.5%)	617 (22.5%)	132 (20.9%)	210 (27.8%)	137 (18.8%)	59 (16.9%)	87 (25.3%)	67 (22.0%)
90k–105k	1,203 (12.4%)	384 (9.7%)	195 (7.5%)	624 (22.7%)	141 (22.3%)	132 (17.5%)	139 (19.1%)	163 (46.6%)	83 (24.1%)	66 (21.6%)
105k+	921 (9.5%)	336 (8.5%)	150 (5.8%)	535 (19.5%)	106 (16.8%)	80 (10.6%)	227 (31.2%)	40 (11.4%)	70 (20.3%)	69 (22.6%)
Unknown	848 (8.8%)	304 (7.7%)	127 (4.9%)	320 (11.7%)	75 (11.9%)	103 (13.6%)	78 (10.7%)	21 (6.0%)	35 (10.2%)	42 (13.8%)
*Urbanization level*										
Central metro (1 M+)	5,395 (55.7%)	2,799 (70.9%)	1,595 (61.6%)	2,194 (80.0%)	442 (69.9%)	685 (90.6%)	645 (88.6%)	161 (46.0%)	225 (65.4%)	278 (91.1%)
Metro (250 K-1 M)	2,125 (22.0%)	823 (20.8%)	535 (20.7%)	468 (17.1%)	150 (23.7%)	63 (8.3%)	77 (10.6%)	160 (45.7%)	98 (28.5%)	24 (7.9%)
Metro	813 (8.4%)	201 (5.1%)	211 (8.1%)	46 (1.7%)	18 (2.8%)	4 (0.5%)	3 (0.4%)	16 (4.6%)	11 (3.2%)	2 (0.7%)
Nonmetro	1,348 (13.9%)	126 (3.2%)	249 (9.6%)	35 (1.3%)	22 (3.5%)	4 (0.5%)	3 (0.4%)	13 (3.7%)	10 (2.9%)	1 (0.3%)

**Table 2:** Patient and tumor characteristics of stomach cancer survivors in South Korea

	Total
	*N* = 204,240
*Development of second primary cancer during follow-up*	
Yes	6,908 (3.4%)
*Vital status*	
No	163,535 (80.1%)
Yes	40,705 (19.9%)
*Follow-up time*	
Mean (SD)	4.6 (± 2.5)
*Sex*	
Female	66,609 (32.6%)
Male	137,631 (67.4%)
*Age at stomach cancer diagnosis*	
<40	5,492 (2.7%)
40–49	20,533 (10.1%)
50–59	48,274 (23.6%)
60–69	59,406 (29.1%)
70–79	51,960 (25.4%)
80+	18,575 (9.1%)
*SEER summary stage of initial stomach cancer*	
Localized	153,945 (75.4%)
Regional	50,295 (24.6%)
*Histological type*	
Diffuse type	40,538 (19.8%)
Intestinal type	154,278 (75.5%)
NOS/Other	9,424 (4.6%)
*Anatomic site*	
Cardia	8,575 (4.2%)
Distal	91,637 (44.9%)
Middle	2,133 (1.0%)
Overlapping/Other	19,151 (9.4%)
Unknown	82,744 (40.5%)
*Received radiotherapy*	
No	203,498 (99.6%)
Yes	742 (0.4%)
*Received chemotherapy*	
No	174,588 (85.5%)
Yes	29,652 (14.5%)
*Received surgery*	
No	20,943 (10.3%)
Yes	183,297 (89.7%)
*Household income* ^a^	
<2	38,828 (19.7%)
3–4	26,324 (12.9%)
5–6	31,231 (15.3%)
7–8	42,082 (20.6%)
9–10	61,960 (30.3%)
Unknown	3,815 (1.9%)
*Urbanization level*	
Capital	34,419 (16.9%)
Metropolitan	52,964 (25.9%)
Nonmetro	116,761 (57.2%)
Unknown	96 (0.0%)

^a^Household income data from Korea were classified into decile groups (1–2, 3–4, 5–6, 7–8, and 9–10), with an additional category for unknown values

**Fig. 1:**
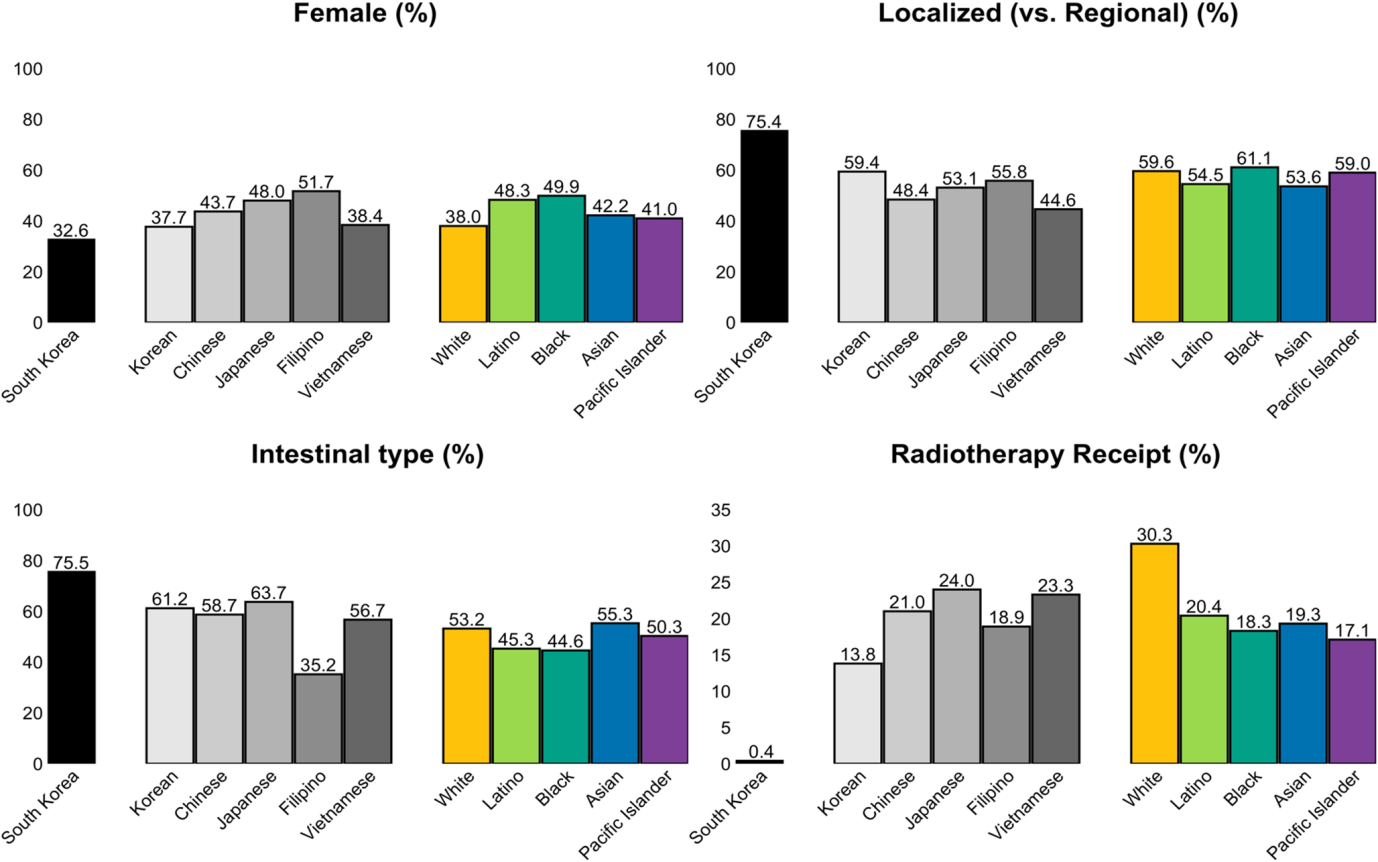
Patient and tumor characteristics at the time of initial stomach cancer diagnosis by race and Asian subgroup in the U.S. and overall in South Korea. Black bars represent South Korean statistics from Cancer Public Library Database (CPLD). Grey bars and colored bars represent U.S. data from the SEER registries, corresponding to Asian Americans and other racial groups, respectively

Figure 1 illustrates key differences in characteristics of stomach cancer patients across racial/ethnic groups in the U.S. and those in South Korea. The proportion of female patients was lowest in the South Korean CPLD cohort (32.6%), in contrast to a broader range observed in U.S. SEER by racial/ethnic group (37.7%–51.7%). Stage at diagnosis also varied: patients in South Korea had the highest proportion diagnosed at a localized stage (75.4%), a pattern similarly observed among Korean American (59.4%) and White (59.6%) patients in the U.S., though to a lesser extent. Histologic subtype distributions revealed that intestinal-type tumors predominated in South Korean patients (75.5%) and were more commonly observed across Asian American subgroups (56.7%–63.7%), compared with White (53.2%), Latino (45.3%), and Black (44.6%) patients. First-course radiotherapy for stomach cancer was rare in South Korea (0.4%) but more common in the U.S. (13.8–30.3%) (Fig. 1).

**Fig. 2:**
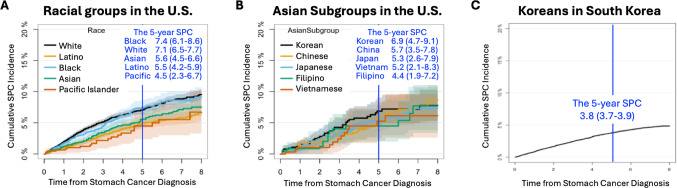
The observed cumulative incidence of SPC in stomach cancer survivors by race and Asian group in the U.S. and overall in South Korea

Cumulative incidence of SPCs showed variation across racial groups (Fig. 2). In the U.S. cohort, the 5-year cumulative incidence was highest among Black (7.4% [95% CI 6.1–8.6]) and White patients (7.1% [95% CI 6.5–7.7]), followed by Asian (5.6% [95% CI 4.5–6.6]), Latino (5.5% [95% CI 4.2–5.9]), and Pacific Islander (4.5% [95% CI 2.3–6.7]) patients, and the difference was statistically significant based on Gray’s test (*p* < 0.001) (Fig. 2A). Although aggregated Asian data showed a moderate SPC incidence (5.6% [95% CI 4.5–6.6]) (Fig. 2A), disaggregated analysis revealed substantial heterogeneity (4.4%-6.9%), highest in Korean (6.9% [95% CI 4.7–9.1]), followed by Chinese (5.7% [95% CI 3.5–7.8]), Japanese (5.3% [95% CI 2.6–7.9]), Vietnamese (5.2% [95% CI 2.1–8.3]), and Filipino (4.4% [95% CI 1.9–7.2]) American patients (Fig. 2B). In comparison, Korean patients in South Korea exhibited the lowest 5-year SPC incidence of 3.8% (95% CI: 3.7–3.9) (Fig. 2C). Lung, breast, and prostate cancers were the most common SPCs in White, Black, and Asian patients, while pancreatic and liver cancers predominated in Hispanic and Pacific Islander patients, with greater diversity observed across Asian subgroups (Supplementary Table 1).

To assess the impact of SPC development on survival, we conducted a Cox model treating SPC as a time-varying covariate. There was association between SPC development and overall mortality (HR = 2.22 [95% CI: 1.96–2.52], *p* < 0.001) (Table 3). Several other covariates showed associations with mortality (Table 3), receiving the first-course surgery treatment (HR = 0.39 [95% CI: 0.37–0.41]), receiving the radiotherapy treatment (HR = 1.13 [95% CI: 1.06–1.12]), or having intestinal (vs. diffuse) type tumors (HR = 0.75 [95% CI: 0.70–0.80]).The corresponding result among stomach cancer survivors in South Korea was consistent with the HR of 1.68 (95% CI: 1.61–1.75) on mortality for those with SPC development compared to those who only had one stomach cancer (Table 3).

**Table 3:** Multivariate Cox analysis of SPC development and overall mortality in stomach cancer survivors in the U.S. and South Korea

	The U.S. SEER database			South Korea Cancer public library database (CPLD)		
	Hazard ratio	95% CI	*P*-value	Hazard ratio	95% CI	*P*-value
SPC development	2.22	1.96–2.52	< 0.001	1.68	1.61–1.75	< 0.001
*Age at initial stomach cancer*						
1. <40	1.00			1.00		
2. 40–49	1.00	0.86–1.21	0.82	1.01	0.93–1.10	0.82
3. 50–59	1.08	0.93–1.26	0.33	1.34	1.24–1.46	< 0.001
4. 60–69	1.20	1.04–1.40	0.01	1.99	1.83–2.15	< 0.001
5. 70–79	1.46	1.26–1.70	< 0.001	3.57	3.30–3.87	< 0.001
6. 80+	2.49	2.13–2.91	< 0.001	6.54	6.02–7.10	< 0.001
*SEER stage of stomach cancer*						
Localized	1.00			1.00		
Regional	2.19	2.06–2.33	< 0.001	2.33	2.28–2.38	< 0.001
*Race*						
White	1.00					
Latino	1.10	1.02–1.18	0.01			
Black	1.08	0.99–1.17	0.07			
Asian	0.92	0.85–0.99	0.03			
Pacific islander	0.79	0.67–0.93	0.00			
*Received surgery*						
No	1.00			0.46	0.45–0.47	< 0.001
Yes	0.39	0.37–0.41	< 0.001			
*Received chemotherapy*						
No	1.00			1.00	1.46–1.55	< 0.001
Yes	1.13	1.06–1.20	< 0.001	1.50		
*Received radiotherapy*						
No	1.00			1.00	0.98–1.25	0.09
Yes	1.13	1.06–1.21	< 0.001	1.11		
*Household income* ^a^						
<60k	1.00			1.00		
60k–75k	0.92	0.84–1.01	0.08	0.87	0.82–0.91	< 0.001
75k–90k	0.87	0.79–0.97	0.01	0.83	0.79–0.88	< 0.001
90k–105k	0.83	0.74–0.92	< 0.001	0.80	0.76–0.85	< 0.001
105k+	0.79	0.69–0.89	< 0.001	0.75	0.72–0.79	< 0.001
Unknown	0.76	0.67–0.86	< 0.001	0.58	0.53–0.63	< 0.001
*Urbanization level*						
Central metro (1 M+)	1.00			1.00		
Metro (250 K-1 M)	1.03	0.97–1.10	0.30	1.04	1.01–1.08	0.02
Metro	1.09	0.97–1.22	0.16	1.02	0.99–1.05	0.21
Nonmetro	1.09	0.98–1.21	0.11	1.38	0.95–1.99	0.09
*Histological type*						
Diffuse type	1.00			1.00		
Intestinal type	0.75	0.70–0.80	< 0.001	0.85	0.82–0.87	< 0.001
NOS/Other	0.36	0.33–0.39	< 0.001	0.79	0.74–0.83	< 0.001
*Anatomic site*						
Cardia	1.00			1.00		
Distal	0.96	0.89–1.04	0.36	0.80	0.77–0.84	< 0.001
Middle	0.92	0.85–0.99	0.03	0.85	0.76–0.95	0.01
Overlapping/Other	1.13	1.03–1.23	0.01	0.96	0.91–1.01	0.12
Unknown				0.83	0.79–0.87	< 0.001

^a^Household income data from Korea were classified into decile groups (1–2, 3–4, 5–6, 7–8, and 9–10), with an additional category for unknown values

**Fig. 3:**
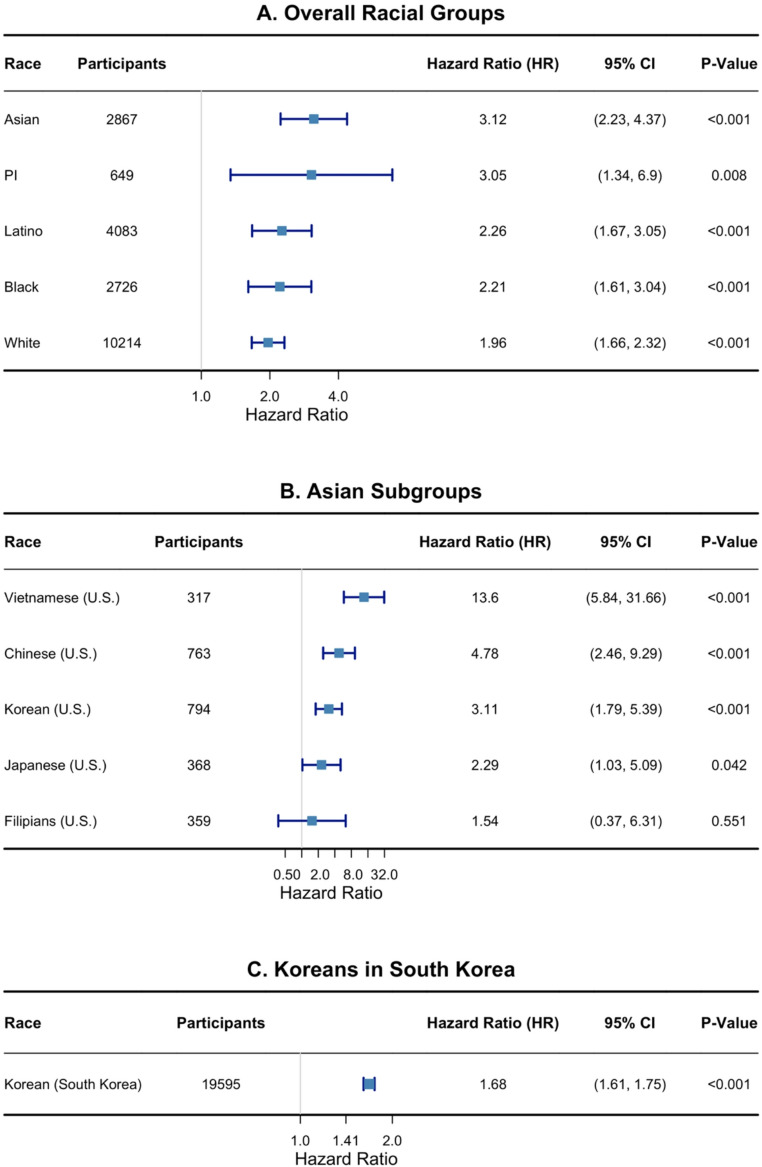
Association between SPC development and overall mortality by race and Asian subgroup among stomach cancer survivors in the U.S. and South Korea

We next evaluated whether the association between SPC development and overall mortality differed by race/ethnicity and country. In stratified analyses (Fig. 3A), Asian American patients exhibited the highest impact of SPC development on mortality (HR = 3.12 [95% CI: 2.23–4.37]), followed by Latinos (HR = 2.26 [95% CI: 1.67–3.05]), Black patients (HR = 2.21 [95% CI: 1.61–3.04]), and White patients (HR = 1.696 [95% CI: 1.66–2.32]). Pacific Islander patients showed a similarly elevated hazard (HR = 3.05 [95% CI: 1.34–6.9]), although the limited number of SPC events in this group (*n* = 21; Table 1) warrants cautious interpretation. These differences in hazard ratios were proved to be marginally statistically different in likelihood ratio test (interaction *p* = 0.11). Difference in the magnitude of the association between SPC and mortality may be partially explained by variations in the most common types of SPCs—such as lung cancer being predominant among Asian American patients (Supplementary Table 1)—as well as by difference in stage and tumor size at SPC, with Black and Latino patients presenting with late-stage and larger SPC (Supplementary Table 2).

Among Asian subgroups, Vietnamese American patients had the highest mortality risk following SPC (HR = 13.6 [95% CI: 5.84–31.66], *p* < 0.001) (Fig. 3B), likely influenced by a greater proportion of late-stage SPCs (Supplementary Table 2). Chinese American patients also demonstrated elevated mortality risk (HR = 4.78 [95% CI: 2.46–9.29], *p* < 0.001), which may be related to much older age at SPC diagnosis than other groups (Mean = 76.1, Supplementary Table 2). Followed by them, Korean American patients showed moderate mortality risk (HR = 3.11 [95% CI: 1.79–5.39], *p* < 0.001). Conversely, Japanese American patients had the lowest observed risk (HR = 2.29 [95% CI: 1.03–5.09], *p* = 0.042), which may reflect SPC detection at earlier stage (Fig. 3B; Supplementary Table 2). These differences in hazard ratios by Asian subgroups were statistically different (interaction *P* = 0.04). Korean patients in South Korea exhibited a comparatively lower hazard ratio for SPC-related mortality (HR = 1.68 [95% CI: 1.61–1.75], *p* < 0.001), which may be explained by their younger age at SPC diagnosis and a lower proportion of distant-stage SPCs (Supplementary Table 3).

## Discussion

In this large, cross-national study of stomach cancer survivors using population-based cancer registries from the U.S. and South Korea, we found that both the incidence of SPCs and the impact of SPC development on overall mortality varied by race/ethnicity and country. Importantly, the inclusion of disaggregated data among Asian subgroups revealed substantial differences in SPC risk that were obscured when Asian Americans were analyzed as a single group. The SPC risk appeared highest among White and Black patients, but disaggregated analyses showed that Korean and Chinese Americans experienced similarly high SPC incidence. SPC-related mortality was pronounced among Asian patients, with Vietnamese and Chinese American survivors demonstrating the highest risk. Notably, patients in South Korea exhibited both the lowest cumulative SPC incidence and a reduced magnitude of SPC-related mortality risk, relative to U.S. populations. Exploratory analyses suggest that tumor etiology (e.g., *H. pylori*-related cancers), radiotherapy, and surveillance practices may partly explain observed differences.

This cross-national analysis revealed both shared and divergent characteristics of primary stomach cancer across racial/ethnic groups in the U.S. and patients in South Korea. Notably, Asian American subgroups and Korean patients in South Korea exhibited several common clinical features at diagnosis, including older age, male predominance, and a higher prevalence of intestinal-type histology—patterns not observed among White, Black, or Latino patients. The observed similarities among Asian populations may reflect shared underlying etiologic factors, such as genetic predisposition, dietary habits, or *H. pylori*–related gastric carcinogenesis. In contrast, differences emerged in stage at diagnosis: while stage distribution among racial/ethnic groups in the U.S. was relatively comparable, Korean patients in South Korea had markedly earlier detection, likely reflecting the impact of the country’s National Cancer Screening Program, [33, 34] which provides biennial endoscopic screening for adults aged 40 and above. Treatment patterns also varied by race/ethnicity and country, particularly in the use of radiotherapy. These findings suggest that while tumor biology may align more closely among Asian populations, differences in cancer detection and treatment are largely shaped by healthcare systems and clinical practice patterns.

These observed differences of primary stomach cancer characteristics and care patterns likely contribute to the variation in subsequent SPC risk. In our analysis, SPC incidence was highest among White and Black patients in the U.S., lower among Asian Americans, and lowest among Korean patients in South Korea. One possible explanation is that *H. pylori*–driven gastric cancer, which is more common in Asian populations, [35–37] may not elevate non-gastric SPC risk. In contrast, other groups (e.g., White, Black patients) may have greater exposure to risk factors like diet, genetics, or smoking, which could raise their risk for non-gastric SPCs. A second contributing factor may be treatment-related effects, particularly exposure to radiotherapy, which has long been associated with an increased risk of subsequent malignancies. [5, 38–43] Ionizing radiation induces DNA damage and long-term genomic instability, processes that can promote carcinogenesis over extended latency periods. [42] In addition, radiotherapy may result in incidental radiation exposure to surrounding normal tissues, a mechanism that has been proposed to contribute to SPC development, [40–42] while the causal relationship among stomach cancer survivors warrants further investigation. In South Korea, stomach cancer is usually treated with surgery and extended lymphadenectomy, with radiotherapy rarely used. [44] Radiotherapy use was only 0.4% in the South Korean data used in this analysis but was common in the U.S. (13.8%–30.3% by race)—consistent with other national data from U.S. National Cancer Database. [45, 46] Furthermore, our exploratory analysis (Supplementary Fig. 2) further showed higher SPC incidence among patients who received radiotherapy. These international findings suggest several hypotheses about the causes and treatment factors influencing racial and geographic differences in SPC risk, highlighting the importance of further research to uncover the underlying biology and develop tailored health system strategies for managing SPC risk.

The development of SPCs also affects overall mortality among stomach cancer survivors, with impacts varying by race/ethnicity and county. While these findings should be interpreted cautiously given the multiple unmeasured factors influencing SPC-related mortality, differences in SPC-related mortality may in part stem from SPC type, tumor characteristics (stage, size), and age at diagnosis. In our cohort, Asian patients had the highest SPC-driven mortality risk, followed by Black patients. Higher mortality in Black patients may result from advanced-stage SPC at diagnosis and delays in treatment, reflecting challenges in timely care. Black patients in the U.S. were more often diagnosed at advanced stages, face delays in diagnosis and treatment, and encounter barriers to ongoing surveillance and care. [47] For Asian Americans, higher SPC mortality is likely linked to older age at diagnosis, which brings greater physiological vulnerability and more comorbidities. Also, this may reflect Asian American survivors’ longer survival after stomach cancer, allowing more SPCs to develop, underscoring the need for ongoing surveillance. Among Asian subgroups, Chinese American patients showed the highest SPC-related mortality, possibly due to older age at diagnosis which may limit the feasibility or effectiveness of aggressive SPC treatment, while Vietnamese patients’ higher mortality may relate to more distant-stage SPCs, suggesting delayed detection. Finally, when comparing stomach cancer survivors who are Korean Americans in the U.S. with Koreans in South Korea, SPC-related mortality was much lower in South Korea. This observation suggests potential system- and country-level difference, such as higher cancer screening rates. We found that the most common SPC types—breast, lung, prostate, and colorectal cancers—were similar across races/ethnicities and countries, suggesting post-stomach cancer screening may aid early detection of SPCs. In recent nationally representative surveys in South Korea, the rates of adhering to screening recommendations for breast, lung, and colorectal cancers have been 72.7%, 58.6%, and 70.7%, respectively. [48] While registry-linked studies are needed to confirm screening’s impact on SPC mortality, our findings highlight the importance of using cancer control resources to improve long-term survivor outcomes. This aligns with the NCI perspective that the cancer care continuum is not linear, but rather circular—cycling through prevention, detection, diagnosis, treatment, and returning to prevention to address multiple primaries, recurrences, and ongoing care.

This study has several strengths. First, it uses two large, national cancer registry data with long-term follow-up, enabling robust estimation of SPC burden and mortality in the U.S. and South Korea. Second, by disaggregating Asian subgroups separately—a first approach in SPC studies of stomach cancer survivors—we revealed substantial heterogeneity in SPC incidence and mortality within these groups. Third, the international comparison of stomach cancer survivors in U.S. and Korea offers valuable insight into how environmental and healthcare system factors influence SPC outcomes. However, several limitations should be noted. First, despite the use of adjusted models, unmeasured confounding—especially from lifestyle factors and *H. pylori* infection, as well as structural factors such as screening practices, healthcare access, and early detection rates across race and country, which were not captured in our data—may obscure a direct causal relationship. Second, differences in SPC definitions across registries could introduce misclassification bias, varying by institution and country, though we applied standardized definitions to enhance comparability. Also, some Asian subpopulations, such as Indian and Pakistani patients in the U.S. SEER, were not included in the subgroup analyses due to limited sample sizes. Lastly, first-course treatment data in SEER and the Korean CPLD were mostly limited to the first 4–6 months after diagnosis, which may not completely reflect all treatments received.

In summary, this study reveals notable differences in SPC incidence among stomach cancer survivors across racial/ethnic groups, particularly within Asian subgroups, and between countries. Furthermore, elevated SPC-driven mortality was found among Asian American and Black patients in the U.S., while rates were much lower in South Korea. The comparison between U.S. and South Korea identified several potential contributing factors for these differences in SPC risk and SPC-related mortality, such as primary tumor etiology, radiotherapy use, and post-treatment surveillance, showing how biology, treatment, and healthcare systems influence long-term outcomes. These findings emphasize the importance of tailored risk assessment for SPC, enhanced post-treatment cancer screening, and personalized management strategies for older survivors. Effective SPC management benefits from addressing both clinical risk factors and healthcare system differences, with a focus on early detection and improving care access for vulnerable groups.

## Supplementary Information

Below is the link to the electronic supplementary material.

Supplementary file1 (DOCX 464 KB)

## Data Availability

The data underlying this article are available in SEER Database: Incidence-Based Mortality-SEER Research Data at https://seer.cancer.gov/data/access.html, and can be accessed with Surveillance Research Program, National Cancer Institute SEER*Stat software (seer.cancer.gov/seerstat). The Cancer Public Library Database (CPLD) in South Korea can be assessed through the K-CURE portal (https:/k-cure.mohw.go.kr). Researchers are required to submit a study proposal with ethical approval from their Institutional Review Board. These requirements must be approved by the National Cancer Data Center (NCDC) review committee before data access is granted.
