# Supplementary material for: Racial/ethnic and geographic differences in second primary cancers in stomach cancer survivors: a comparative study of U.S. and South Korea
Source: Gastric Cancer. 2026 Mar 9;29(3):506–18. doi: 10.1007/s10120-026-01728-9 (PMC13124850; doi:10.1007/s10120-026-01728-9)
Supplement: Supplementary file 1 — Supplementary file1 (DOCX 464 KB) [file 10120_2026_1728_MOESM1_ESM.docx]

**Supplementary Files**

**Supplementary Method 1.** Details of study databases

**Supplement Figure 1.** Flow diagram of study population selection in the SEER registries in the U.S. and the Cancer Public Library Database in South Korea

**Supplement Figure 2.** The observed cumulative incidence of SPC in stomach cancer survivors by radiotherapy in the U.S.

**Supplement Table 1**. Top 5 most common types of SPC in stomach cancer survivors by race and Asian subgroup

**Supplement Table 2.** Patient and tumor characteristics of SPC by race and Asian subgroups

**Supplement Table 3.** Patient and tumor characteristics of SPC in stomach cancer survivors in South Korea

**Supplementary Methods**

**Supplementary Method 1.** Details of study databases

This study utilized two national cancer registries. First is from the U.S. SEER Program-17, assessed via the National Cancer Institute’s SEER*Stat software and the second is from the Cancer Public Library Database (CPLD) in South Korea (1).

The U.S. SEER-17 collects high-quality, population-based cancer incidence and survival data from 17 U.S geographic regions, representing approximately 26.5% of the national population.

The CPLD is a nationwide cohort of Korean cancer patients, launched in 2022 under the Korea-Clinical Data Utilization network for Research Excellence Initiative. It integrates data from several national sources, including the Korean Central Cancer Registry, Cause of Death Database, National Health Information Database, and the National Health Insurance Service Research Database. By the 2023 release, the annually updated dataset includes around 1.98 million cancer cases diagnosed (2012 –2019). It offers mortality follow-up through 2020, longitudinal claims and screening data through 2021, and detailed information on tumor site, morphology, SEER stage, healthcare use, and behavioral risk factors (2).

These two databases provide basic patient demographics, tumor characteristics, first-course treatment modalities, and survival outcomes.

**Supplementary Figures**

**Supplementary Figure 1.** Flow diagram of study population selection in the SEER registries in the U.S. and the Cancer Public Library Database (CPLD) in South Korea


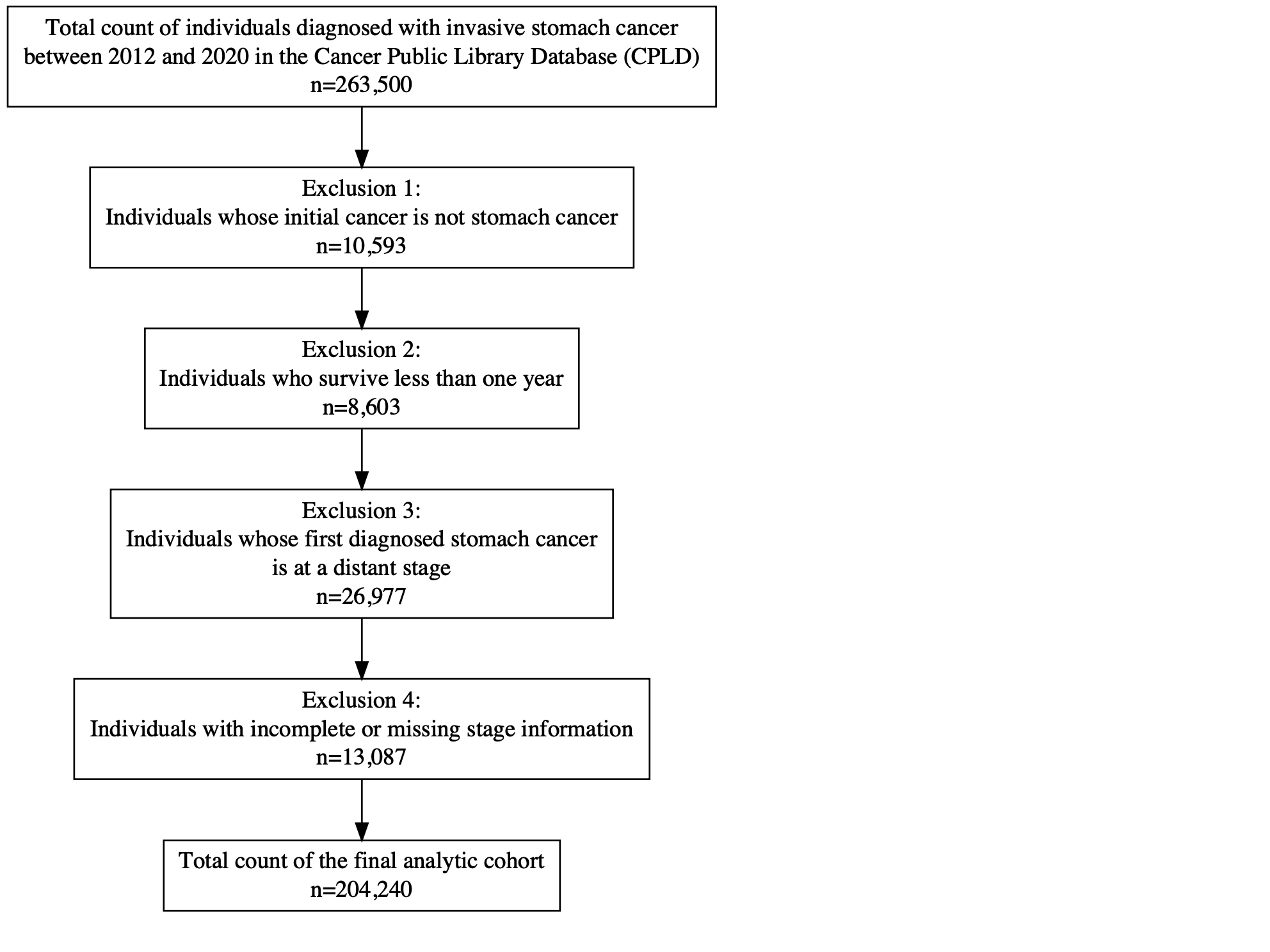

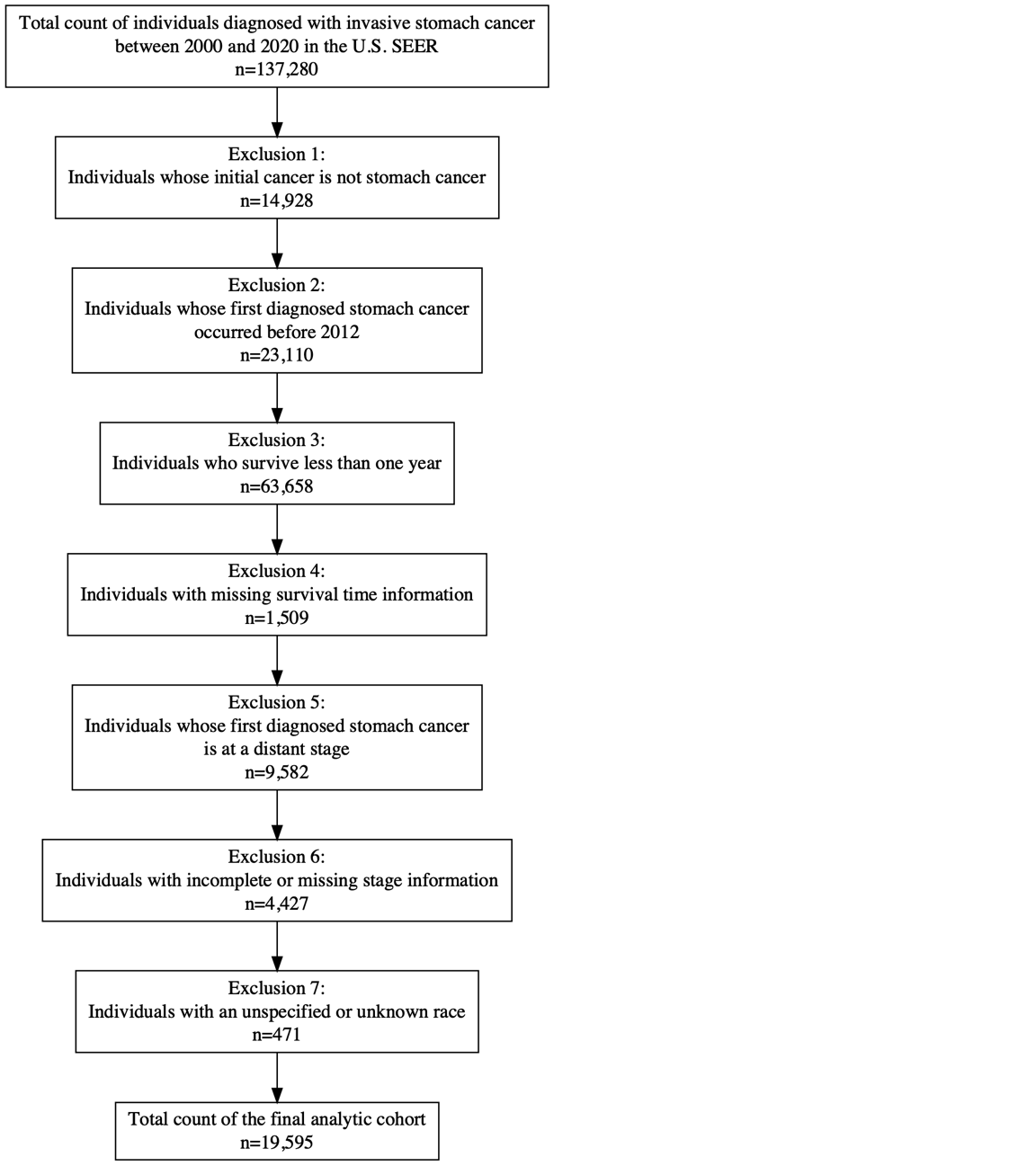


**A**

**B**

**Supplementary Figure 2.** The observed cumulative incidence of SPC in stomach cancer survivors by radiotherapy in the U.S.

**B**

**A**

**5-year survivors**

**All study cohort (+1 survivors)**


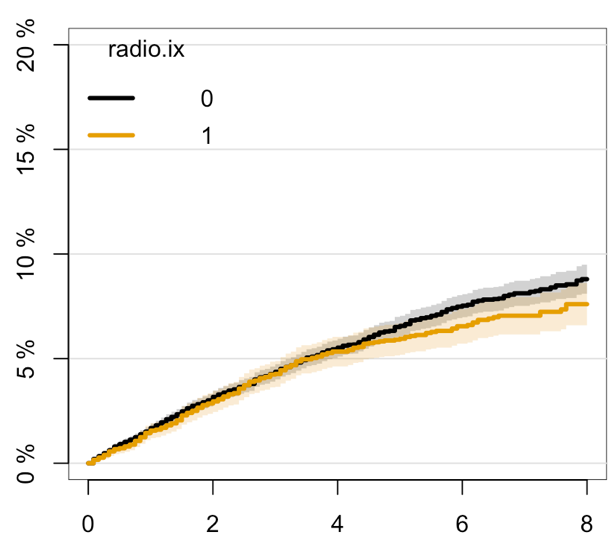

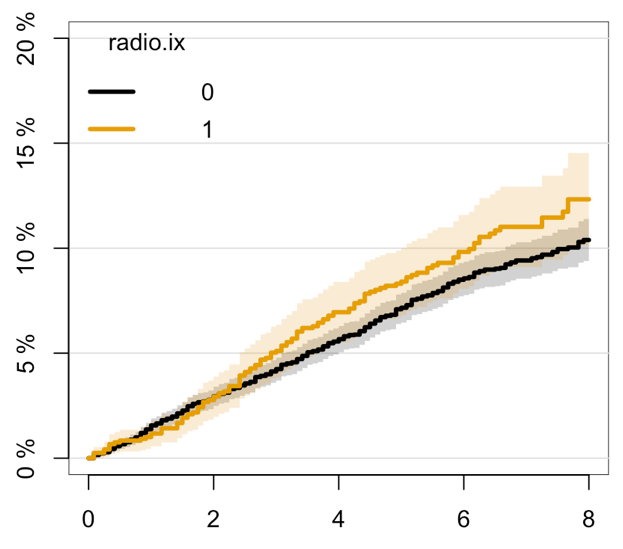


Cumulative SPC incidence

Cumulative SPC incidence

Time from stomach cancer diagnosis (year)

Time from stomach cancer diagnosis (year)

**Supplementary Tables**

**Supplement Table 1.** Top 5 most common types of SPC in stomach cancer survivors by race and Asian subgroup

| Overall Racial Groups | | | | | | | | | | | | | | |
| --- | --- | --- | --- | --- | --- | --- | --- | --- | --- | --- | --- | --- | --- | --- |
| White |  |  | Black |  |  | Hispanic |  |  | Asian |  |  | Pacific |  |  |
| Type | N | (%) | Type | N | (%) | Type | N | (%) | Type | N | (%) | Type | N | (%) |
| Total | 601 | 100.0 | Total | 150 | 100.0 | Total | 150 | 100.0 | Total | 128 | 100.0 | Total | 21 | 100.0 |
| C34 (Lung) | 90 | 15.0 | C61 (Prostate) | 28 | 18.7 | C61 (Prostate) | 20 | 13.3 | C34 (Lung) | 23 | 18.0 | C50 (Breast) | 4 | 19.0 |
| C61 (Prostate) | 86 | 14.3 | C34 (Lung) | 25 | 16.7 | C34 (Lung) | 16 | 10.7 | C50 (Breast) | 16 | 12.5 | C22 (Liver) | 3 | 14.3 |
| C50 (Breast) | 51 | 8.5 | C50 (Breast) | 23 | 15.3 | C25 (Pancreas) | 15 | 10.0 | C61 (Prostate) | 13 | 10.2 | C34 (Lung) | 3 | 14.3 |
| C44 (Skin) | 49 | 8.2 | C42 (Hemato) | 11 | 7.3 | C18 (Colon) | 11 | 7.3 | C42 (Hemato) | 10 | 7.8 | C18 (Colon) | 2 | 9.5 |
| C67 (Bladder) | 41 | 6.8 | C25 (Pancreas) | 9 | 6.0 | C15 (Esophagus) | 9 | 6.0 | C18 (Colon) | 8 | 6.3 | C42 (Hemato) | 1 | 4.8 |
|  |  |  |  |  |  |  |  |  |  |  |  |  |  |  |
| Asian Subgroups | | | | | | | | | | | | | | |
| Korean |  |  | Chinese |  |  | Japanese |  |  | Filipino |  |  | Vietnamese |  |  |
| Type | N | (%) | Type | N | (%) | Type | N | (%) | Type | N | (%) | Type | N | (%) |
| Total | 40 | 100.0 | Total | 35 | 100.0 | Total | 18 | 100.0 | Total | 15 | 100.0 | Total | 12 | 100.0 |
| C34 (Lung) | 7 | 17.5 | C34 (Lung) | 8 | 22.9 | C25 (Pancreas) | 4 | 22.2 | C50 (Breast) | 4 | 26.7 | C20 (Rectum) | 2 | 16.7 |
| C18 (Colon) | 5 | 12.5 | C50 (Breast) | 4 | 11.4 | C34 (Lung) | 4 | 22.2 | C15 (Esophagus) | 2 | 13.3 | C34 (Lung) | 2 | 16.7 |
| C67 (Bladder) | 5 | 12.5 | C18 (Colon) | 3 | 8.6 | C61 (Prostate) | 4 | 22.2 | C34 (Lung) | 2 | 13.3 | C42 (Hemato) | 2 | 16.7 |
| C42 (Hemato) | 4 | 10.0 | C15 (Esophagus) | 2 | 5.7 | C50 (Breast) | 3 | 16.7 | C61 (Prostate) | 2 | 13.3 | C12 (Pyriform) | 1 | 8.3 |
| C50 (Breast) | 4 | 10.0 | C17 (Small intestine) | 2 | 5.7 | C32 (Larynx) | 1 | 5.6 | C20 (Rectum) | 1 | 6.7 | C24 (Biliary tract) | 1 | 8.3 |
|  |  |  |  |  |  |  |  |  |  |  |  |  |  |  |
| Koreans in South Korea | |  |  |  |  |  |  |  |  |  |  |  |  |  |
| Type | N | (%) |  |  |  |  |  |  |  |  |  |  |  |  |
| Total | 6908 | 100.0 |  |  |  |  |  |  |  |  |  |  |  |  |
| C34 (Lung) | 1365 | 19.8 |  |  |  |  |  |  |  |  |  |  |  |  |
| C61 (Prostate) | 838 | 12.1 |  |  |  |  |  |  |  |  |  |  |  |  |
| C22 (Liver) | 579 | 8.4 |  |  |  |  |  |  |  |  |  |  |  |  |
| C18 (Colon) | 531 | 7.7 |  |  |  |  |  |  |  |  |  |  |  |  |
| C25 (Pancreas) | 306 | 4.4 |  |  |  |  |  |  |  |  |  |  |  |  |

**Supplement Table 2.** Patient and tumor characteristics of SPC by race and Asian subgroups

| Variables | Overall Racial Groups | | | | | | P-value |
| --- | --- | --- | --- | --- | --- | --- | --- |
|  | Total | White | Latino | Black | Asian | Pacific Islander |  |
|  | No. 1,050 | No. 601 | No. 150 | No. 150 | No. 128 | No. 21 |  |
| Age at Diagnosis | |  |  |  |  |  |  |
| Mean (SD) | 70.3 (±10.9) | 70.6 (±10.7) | 68.9 (±12.9) | 68.4 (±9.9) | 73.4 (±9.7) | 65.3 (±10.4) | 0.0002 |
| Grouped Age at Diagnosis | | |  |  |  |  |  |
| 1.<40 | 9 (0.9%) | 5 (0.8%) | 3 (2.0%) | 0 (0.0%) | 1 (0.8%) | 0 (0.0%) | 0.002 |
| 2.40-90 | 34 (3.2%) | 14 (2.3%) | 11 (7.3%) | 5 (3.3%) | 1 (0.8%) | 3 (14.3%) |  |
| 3.50-59 | 119 (11.3%) | 67 (11.1%) | 19 (12.7%) | 22 (14.7%) | 9 (7.0%) | 2 (9.5%) |  |
| 4.60-69 | 289 (27.5%) | 169 (28.1%) | 36 (24.0%) | 49 (32.7%) | 27 (21.1%) | 8 (38.1%) |  |
| 5.70-79 | 384 (36.6%) | 219 (36.4%) | 51 (34.0%) | 54 (36.0%) | 53 (41.4%) | 7 (33.3%) |  |
| 6.80+ | 215 (20.5%) | 127 (21.1%) | 30 (20.0%) | 20 (13.3%) | 37 (28.9%) | 1 (4.8%) |  |
| Cancer Stage | |  |  |  |  |  |  |
| 0.In situ | 84 (8.0%) | 59 (9.8%) | 3 (2.0%) | 7 (4.7%) | 11 (8.6%) | 4 (19.0%) | 0.022 |
| 1.Localized | 498 (47.4%) | 280 (46.6%) | 73 (48.7%) | 76 (50.7%) | 58 (45.3%) | 11 (52.4%) |  |
| 2.Regional | 187 (17.8%) | 101 (16.8%) | 32 (21.3%) | 26 (17.3%) | 26 (20.3%) | 2 (9.5%) |  |
| 3.Distant | 188 (17.9%) | 102 (17.0%) | 24 (16.0%) | 33 (22.0%) | 25 (19.5%) | 4 (19.0%) |  |
| 4.NA | 93 (8.9%) | 59 (9.8%) | 18 (12.0%) | 8 (5.3%) | 8 (6.2%) | 0 (0.0%) |  |
| Tumor Size | |  |  |  |  |  |  |
| 1.<2cm | 231 (22.0%) | 141 (23.5%) | 29 (19.3%) | 34 (22.7%) | 23 (18.0%) | 4 (19.0%) | 0.13 |
| 2.<4cm | 224 (21.3%) | 118 (19.6%) | 35 (23.3%) | 26 (17.3%) | 34 (26.6%) | 11 (52.4%) |  |
| 3.<6cm | 106 (10.1%) | 48 (8.0%) | 20 (13.3%) | 17 (11.3%) | 18 (14.1%) | 3 (14.3%) |  |
| 4.<10cm | 51 (4.9%) | 32 (5.3%) | 6 (4.0%) | 10 (6.7%) | 3 (2.3%) | 0 (0.0%) |  |
| 5.10+ | 26 (2.5%) | 13 (2.2%) | 3 (2.0%) | 5 (3.3%) | 5 (3.9%) | 0 (0.0%) |  |
| Missing | 412 (39.2%) | 249 (41.4%) | 57 (38.0%) | 58 (38.7%) | 45 (35.2%) | 3 (14.3%) |  |
| Time to Treatment (Months) | | |  |  |  |  |  |
| Mean (SD) | 1.3 (±1.6) | 1.2 (±1.5) | 1.5 (±1.7) | 1.7 (±2.0) | 1.5 (±1.6) | 1.1 (±1.0) | 0.03 |
| Missing | 280 (26.7%) | 157 (26.1%) | 56 (37.3%) | 39 (26.0%) | 27 (21.1%) | 1 (4.8%) |  |
| Variables | Asian Subgroups | | | | | | P-value |
|  | Total | Korean | Chinese | Japanese | Filipino | Vietnamese |  |
|  | No. 120 | No. 40 | No. 35 | No. 18 | No. 15 | No. 12 |  |
| Age at Diagnosis | |  |  |  |  |  |  |
| Mean (SD) | 73.9 (±9.1) | 73.7 (±8.8) | 76.1 (±9.2) | 76.3 (±9.7) | 70.3 (±6.7) | 69.1 (±8.8) | 0.056 |
| Grouped Age at Diagnosis | | |  |  |  |  |  |
| 1.<40 | 0 (0.0%) | 0 (0.0%) | 0 (0.0%) | 0 (0.0%) | 0 (0.0%) | 0 (0.0%) | 0.3 |
| 2.40-90 | 1 (0.8%) | 1 (2.5%) | 0 (0.0%) | 0 (0.0%) | 0 (0.0%) | 0 (0.0%) |  |
| 3.50-59 | 7 (5.8%) | 2 (5.0%) | 1 (2.9%) | 1 (5.6%) | 1 (6.7%) | 2 (16.7%) |  |
| 4.60-69 | 26 (21.7%) | 5 (12.5%) | 9 (25.7%) | 4 (22.2%) | 5 (33.3%) | 3 (25.0%) |  |
| 5.70-79 | 50 (41.7%) | 20 (50.0%) | 10 (28.6%) | 7 (38.9%) | 7 (46.7%) | 6 (50.0%) |  |
| 6.80+ | 36 (30.0%) | 12 (30.0%) | 15 (42.9%) | 6 (33.3%) | 2 (13.3%) | 1 (8.3%) |  |
| Cancer Stage | |  |  |  |  |  |  |
| 0.In situ | 11 (9.2%) | 4 (10.0%) | 1 (2.9%) | 3 (16.7%) | 2 (13.3%) | 1 (8.3%) | 0.85 |
| 1.Localized | 54 (45.0%) | 18 (45.0%) | 16 (45.7%) | 8 (44.4%) | 8 (53.3%) | 4 (33.3%) |  |
| 2.Regional | 25 (20.8%) | 7 (17.5%) | 8 (22.9%) | 5 (27.8%) | 3 (20.0%) | 2 (16.7%) |  |
| 3.Distant | 22 (18.3%) | 9 (22.5%) | 7 (20.0%) | 1 (5.6%) | 1 (6.7%) | 4 (33.3%) |  |
| 4.NA | 8 (6.7%) | 2 (5.0%) | 3 (8.6%) | 1 (5.6%) | 1 (6.7%) | 1 (8.3%) |  |
| Tumor Size | |  |  |  |  |  |  |
| 1.<2cm | 21 (17.5%) | 5 (12.5%) | 7 (20.0%) | 4 (22.2%) | 4 (26.7%) | 1 (8.3%) | 0.67 |
| 2.<4cm | 33 (27.5%) | 9 (22.5%) | 11 (31.4%) | 7 (38.9%) | 3 (20.0%) | 3 (25.0%) |  |
| 3.<6cm | 16 (13.3%) | 5 (12.5%) | 6 (17.1%) | 2 (11.1%) | 3 (20.0%) | 0 (0.0%) |  |
| 4.<10cm | 3 (2.5%) | 2 (5.0%) | 0 (0.0%) | 0 (0.0%) | 0 (0.0%) | 1 (8.3%) |  |
| 5.10+ | 5 (4.2%) | 4 (10.0%) | 1 (2.9%) | 0 (0.0%) | 0 (0.0%) | 0 (0.0%) |  |
| Missing | 42 (35.0%) | 15 (37.5%) | 10 (28.6%) | 5 (27.8%) | 5 (33.3%) | 7 (58.3%) |  |
| Time to Treatment (Months) | | |  |  |  |  |  |
| Mean (SD) | 1.5 (±1.7) | 1.6 (±2.0) | 1.4 (±1.2) | 1.4 (±1.2) | 2.1 (±2.3) | 1.2 (±1.4) | 0.73 |
| Missing | 26 (21.7%) | 7 (17.5%) | 8 (22.9%) | 5 (27.8%) | 3 (20.0%) | 3 (25.0%) |  |

**Supplement Table 3.** Patient and tumor characteristics of SPC in stomach cancer survivors in South Korea

|  | Total |
| --- | --- |
|  | N= 6,908 |
| Grouped Age at Diagnosis | |
| 1. <40 | 24 (0.3%) |
| 2. 40-49 | 202 (2.9%) |
| 3. 50-59 | 730 (10.6%) |
| 4. 60-69 | 2,031 (29.4%) |
| 5. 70-79 | 2,841 (41.1%) |
| 6. 80+ | 1,080 (15.6%) |
| Cancer Stage | |
| 0.In situ | 512 (7.4%) |
| 1.Localized | 2,888 (41.8%) |
| 2.Regional | 1,767 (25.6%) |
| 3.Distant | 1,122 (16.2%) |
| 4.Unknown | 619 (9.0%) |
